# Bowel cancer chemoprevention - ready for the clinic?

**DOI:** 10.1186/1897-4287-10-S2-A12

**Published:** 2012-04-12

**Authors:** J Burn

**Affiliations:** 1 Newcastle University, UK

## 

In 1988 Gabriel Kune of Melbourne published a seminal paper reporting a negative association of the use of non-steroidal anti-inflammatory drugs such as aspirin and colorectal cancer. This prompted a deluge of case control and cohort studies, all but one of which confirmed the finding. The conclusion of these observational studies was that regular aspirin users had half as many cancers. Four randomised controlled trials (RCTs) using adenoma prevention as their endpoint gave a more mixed result but meta-analysis supported benefit though at a more modest level. Rothwell and colleagues returned to the cancer registry to look up people who took part in the early vascular disease prevention trials using aspirin and found that about 7 years after starting the trial, those who had been given aspirin started to show a reduced cancer rate and a reduced cancer mortality. The CAPP1 study recruited and followed 133 carriers of Familial Adenomatous Polyposis to test 600mg aspirin and showed a weak effect on adenoma formation and evidence of fewer large polyps. CAPP2 recruited 1009 carriers of Lynch syndrome resulting from a germline defect in a mismatch repair gene, and tested 600mg aspirin and/or 30g Novelose, a resistant starch, over four years. Australia was a key contributor to this international trial. At the end of intervention phase there was no difference in overall colorectal neoplasia in any of the groups. Double blind post trial follow-up has revealed a dramatic effect; at a mean of 5 years from randomisation, those who had been taking the active aspirin have significantly fewer colorectal cancers. Confining analysis to the primary endpoint of colorectal cancer after a minimum of 2 years on aspirin revealed 60% fewer cancers. A similar effect was evident when all Lynch syndrome cancers were considered (paper in press).

All people at risk of Lynch syndrome should be advised to take aspirin and their physicians should take usual measures to reduce the recognised burden of side effects. This should include consideration of eradication of H. pylori and use of a regular proton pump inhibitor if there is evidence of gastric erosion. CAPP3 will compare three different doses of enteric coated aspirin: 100mg, 300mg and 600mg in proven gene carriers between 18 and 60 with treatment for a minimum of 5 years and unlimited follow-up. In our present state of knowledge a pragmatic position is to recommend immediate introduction of a low dose daily aspirin (75-100mg) and encouragement to log on to the new CAPP3 website to keep abreast of the emerging story. This dose will not preclude joining the RCT in 2012 and will allow us to explore methods to personalise the aspirin dose in future by assessment of the genetic basis of aspirin sensitivity and resistance.

